# SLAMF Receptor Expression Identifies an Immune Signature That Characterizes Systemic Lupus Erythematosus

**DOI:** 10.3389/fimmu.2022.843059

**Published:** 2022-05-06

**Authors:** Morgane Humbel, Florence Bellanger, Alice Horisberger, Madeleine Suffiotti, Natalia Fluder, Mariko Makhmutova, Amandine Mathias, Renaud Du Pasquier, Craig Fenwick, Camillo Ribi, Denis Comte

**Affiliations:** ^1^ Service of Immunology and Allergy, Department of Medicine, Lausanne University Hospital and University of Lausanne, Lausanne, Switzerland; ^2^ Machine Learning and Optimization Laboratory, Swiss Federal Institute of Technology in Lausanne, Lausanne, Switzerland; ^3^ Service of Neurology, Department of Clinical Neurosciences, University Hospital of Lausanne and University of Lausanne, Lausanne, Switzerland

**Keywords:** SLE - systemic lupus erythematosus, SLAMF, autoimmunity, immune signature, biomarker

## Abstract

Systemic lupus erythematosus (SLE) is a chronic autoimmune disease of unknown etiology, linked to alterations in both the innate and the adaptive immune system. Due to the heterogeneity of the clinical presentation, the diagnosis of SLE remains complicated and is often made years after the first symptoms manifest, delaying treatment, and worsening the prognosis. Several studies have shown that signaling lymphocytic activation molecule family (SLAMF) receptors are aberrantly expressed and dysfunctional in SLE immune cells, contributing to the altered cellular function observed in these patients. The aim of this study was to determine whether altered co-/expression of SLAMF receptors on peripheral blood mononuclear cells (PBMC) identifies SLE characteristic cell populations. To this end, single cell mass cytometry and bioinformatic analysis were exploited to compare SLE patients to healthy and autoimmune diseases controls. First, the expression of each SLAMF receptor on all PBMC populations was investigated. We observed that SLAMF1+ B cells (referred to as SLEB1) were increased in SLE compared to controls. Furthermore, the frequency of SLAMF4+ monocytes and SLAMF4+ NK were inversely correlated with disease activity, whereas the frequency SLAMF1+ CD4+ TDEM cells were directly correlated with disease activity. Consensus clustering analysis identified two cell clusters that presented significantly increased frequency in SLE compared to controls: switch memory B cells expressing SLAMF1, SLAMF3, SLAMF5, SLAMF6 (referred to as SLESMB) and circulating T follicular helper cells expressing the same SLAMF receptors (referred to as SLEcTFH). Finally, the robustness of the identified cell populations as biomarkers for SLE was evaluated through ROC curve analysis. The combined measurement of SLEcTFH and SLEB1 or SLESMB cells identified SLE patients in 90% of cases. In conclusion, this study identified an immune signature for SLE based on the expression of SLAMF receptors on PBMC, further highlighting the involvement of SLAMF receptors in the pathogenesis of SLE.

## Introduction

Systemic lupus erythematosus (SLE) is a chronic inflammatory heterogenous autoimmune disease that mostly affects women of childbearing age (1). Over the past decade, great strides have been made in understanding the pathogenesis of the disease. However, the etiology remains unidentified, making the development of new diagnostic tests and therapeutic approaches challenging.

In recent years, research has focused on identifying novel biomarkers for SLE. Most of the suggested biomarkers are proteins involved in cellular communication, including cytokines, chemokines and growth factors, as well as cell surface receptors (2, 3). From this point of view, signaling lymphocytic activation molecule family (SLAMF) receptors are type I glycoprotein surface receptors expressed on all hematopoietic cells (4). This receptor family includes nine members: SLAMF1 (CD150 or SLAM), SLAMF2 (CD48), SLAMF3 (CD229 or Ly9), SLAMF4 (CD244 or 2B4), SLAMF5 (CD84), SLAMF6 (CD352, NTBA or SF2000 in human or Ly108 in mice), SLAMF7 (CD319, CS1 or CRACC), SLAMF8 (CD353 or BLAME) and SLAMF9 (CD84-H1 or SF2001). SLAMF receptors represent a complex system implicated in cell-to-cell contact and cell activation. They have the unique property of being self-ligands (except for SLAMF2 and SLAMF4 that bind each other) and they can act as a ligand or a receptor depending on the cell by which they are expressed (5). Each hematopoietic cell expresses three to five different SLAMF molecules and they signal *via* recruitment of adaptor proteins to provide a co-stimulatory or co-inhibitory message that influences cell activation (5). Genome wide association studies have identified that SLAMF receptors are located in the 1q23 locus on chromosome 1, which was identified as a susceptibility locus for SLE (6). Furthermore, various studies have evaluated the alteration of SLAMF expression and function in peripheral blood mononuclear cells (PBMC) from SLE patients (7–20). From this point of view, recent important findings concern SLAMF1, SLAMF3, SLAMF4 and SLAMF7. SLAMF1 has been shown to be overexpressed on the surface of T and B cells isolated from SLE patients (8, 17). In addition, this receptor appears to play a key role in the interaction between T and B cells, as *in vitro* data suggest that the binding of SLAMF1 with a specific monoclonal antibody inhibits the interaction between these cell populations, and thus suppresses B cell differentiation (8). In another study, the authors showed that SLAMF3 plays a role in the differentiation of regulatory T cells, through the enhancement of CD4+ T cells’ sensitivity to IL-2, a cytokine whose availability is reduced in patients with SLE (10). Furthermore, SLAMF4 and SLAMF7 are two surface receptors that have been shown to be highly expressed on cytotoxic cells (17). Their expression is altered on SLE CD8+ T cells and NK cells (13, 15, 18–20). Data on SLE CD8+ T cells and NK cells has shown that the engagement of SLAMF7 with specific monoclonal antibodies promotes their cytotoxic function (degranulation and cytokines production) (15, 16). Other findings on SLAMF receptors in SLE are summarized in our recent review (4).

So far, most studies on SLAMF receptors in SLE focused on one receptor at a time and few data examined the co-expression of multiple SLAMF receptors at a single cell level (4).

In this research project, single cell mass cytometry was exploited to perform in-depth immunophenotyping of SLE PBMC to determine the expression of all SLAMF receptors at single cell level. The pattern of expression of SLAMF receptors was compared to healthy and autoimmune diseases controls. We hypothesize that the altered pattern of expression of SLAMF receptors on PBMC contribute to the impaired cell activation and cell-to-cell contact that lead to the development of autoimmunity. Accordingly, SLAMF receptor expression patterns define a SLE specific immune signature.

## Materials and Methods

### Cohorts

SLE patients were diagnosed according to the American College of Rheumatology classification criteria and/or the Systemic Lupus International Collaborating Clinics (SLICC) criteria  (21, 22), and were recruited from the Division of Immunology and Allergy at Centre Hospitalier Universitaire Vaudois (CHUV). Current or past use of belimumab or rituximab was an exclusion criterion. All patients and controls were included in the Swiss Systemic Lupus Erythematosus Cohort Study (SSCS) (23). Disease activity score was measured using the SLE Disease Activity Index (SLEDAI) scoring system. We categorized patients into three groups of disease activity: inactive (SLEDAI 0-3), moderate (SLEDAI 4-10) and active (SLEDAI >10).

Two distinct cohorts were examined: cohort 1 included 28 SLE patients and age-, sex-, and ethnicity-matched healthy controls (**Supplementary Table 1A**). Cohort 2 included 10 patients with SLE, 10 age-, sex-, and ethnicity-matched healthy controls, 10 patients with biopsy-proven sarcoidosis (SAR), 10 patients with Sjögren’s syndrome (SJS; based on the 2002 American-European Classification Criteria) and 10 patients with multiple sclerosis (MS; based on the 2017 McDonald Criteria) (**Supplementary Table 1B**). For MS patients, treatment with corticosteroids within three months before the blood draw was an exclusion criterion.

### Cell Isolation

Analysis of absolute cell count was performed on fresh blood by flow cytometry according to standard diagnostic measurements.

For mass cytometry analysis, peripheral blood mononuclear cells (PBMC) were isolated by density gradient centrifugation (FICOLL 400, Merck, Switzerland), from 22.5 ml peripheral blood, and then cryopreserved in liquid nitrogen.

### Single Cell Mass Cytometry

Samples were stained according to a previously published approach (16). Briefly, cryopreserved PBMC from SLE patients and controls (healthy and autoimmune) were thawed, resuspended in RPMI (completed with 20% heat-inactivated serum). Cells (1 Mio per individual on average) were stained for live/dead with cisplatin 50 µg (5min, room temperature (RT)), barcoded with CD45-metal conjugated antibodies (20min, RT, **Supplementary Tables 2A, B**) and then pooled. For cohort 1, two HC and two SLE were pooled, for cohort 2 one HC, SLE, SAR, SJS, MS sample were pooled in each experiment. Next, cells were incubated with metal conjugated antibody mix for the extracellular staining (20min, RT). The panel consisted of 39 markers, including markers for SLAMF receptors and for the main PBMC populations (CD4+ T cells, CD8+ T cells, double negative T cells (DN), B cells, natural killer (NK) cells, dendritic cells (DC) and monocytes) and differentiated subsets (**Supplementary Table 2C**). Cells were washed and fixed with 2.4% paraformaldehyde (10 min, RT). Labeled samples were acquired on a Helios Cytof System (Fluidigm). For each experiment at least 500’000 cells were acquired per patient. Flow cytometry standard (FCS) files were normalized to EQ Four Element calibration beads using CyTOF software.

### Data Analysis and Statistics

Data were debarcoded on Cytobank software (Beckman Coulter) and fcs files were generated. The fcs files were then analyzed using FlowJo™ software (version 10.2, Becton, Dickinson and Company). All major PBMC populations and subpopulations were gated according to the gating strategy presented in **Supplementary Figure 1**. The data were then processed using GraphPad prism (version 8), R software (version 3.5.1) or Python (version 3.8.5). Statistical analysis was performed with GraphPad prism. Specifications of test exploited and sample size are specified in the figure descriptions. In general, data (cell subset frequencies) were transformed into log_10_(x+1) and normality was assessed with Shapiro-Wilk test. Two groups were compared using Welch’s T test (or Mann-Whitney T test if normality test failed). One-way ANOVA was used for multiple group comparison with normal distribution and p-values were adjusted for multiple testing using Tukey’s test (comparison between all groups) or Dunnett’s test (comparison to a control group). Correlations were assessed using Pearson’s correlation. All data are presented as mean “±” standard error of the mean (SEM). A p-value ≤ 0.05 was considered statistically significant.

Manually gated cell sub-/populations were imported in R studio environment and processed as previously described (16). Briefly, single cell expression was transformed using hyperbolic inverse sine (with cofactor 5) (24). Dimensionality reduction and 2-dimensional visualization were done using the Barnes-Hut implementation of t-stochastic neighboring embedding algorithm (Rtsne package). Unsupervised clustering analysis was performed on previously gated PBMC using self-organizing map in combination with consensus clustering (FlowSOM package). The parameters used for clustering were SLAMF1, SLAMF3, SLAMF4, SLAMF5, SLAMF6 and SLAMF7. The analysis was repeated on subpopulations of cells to ensure consistency of findings. Manual gating was then performed to confirm the existence of an identified cluster. A minimum of 100 cells was required for a cell subset to be considered for further analysis.

Python (Scikit-Learn library) was used to normalize cell frequencies (min-max normalization) of SLEB1, SLEcTFH and SLESMB population. The normalized frequencies were then summed and averaged to obtain combination of the different measurements. ROC curve analysis was used to determine the ability of these measures to distinguish a patient with SLE from healthy or autoimmune controls. The area under the curve (AUC) represents the accuracy of a measurement in distinguishing SLE from controls, and was therefore used as an indicator of separation between groups. Youden index was used to determine the optimal cut-off to separate SLE patients from controls. This cut-off was then applied to cohort 2 to determine the specificity of the approach in identifying SLE patients among patients affected by other autoimmune diseases.

### Study Approval

Informed written consent was obtained from all participants prior to inclusion and the study was approved by the Institutional Review Board (SwissEthics 2017-01434 and 2018-01622), in compliance with the Declaration of Helsinki.

## Results

### Distribution of PBMC Populations Is Altered in SLE Patients

**Figure 1:**
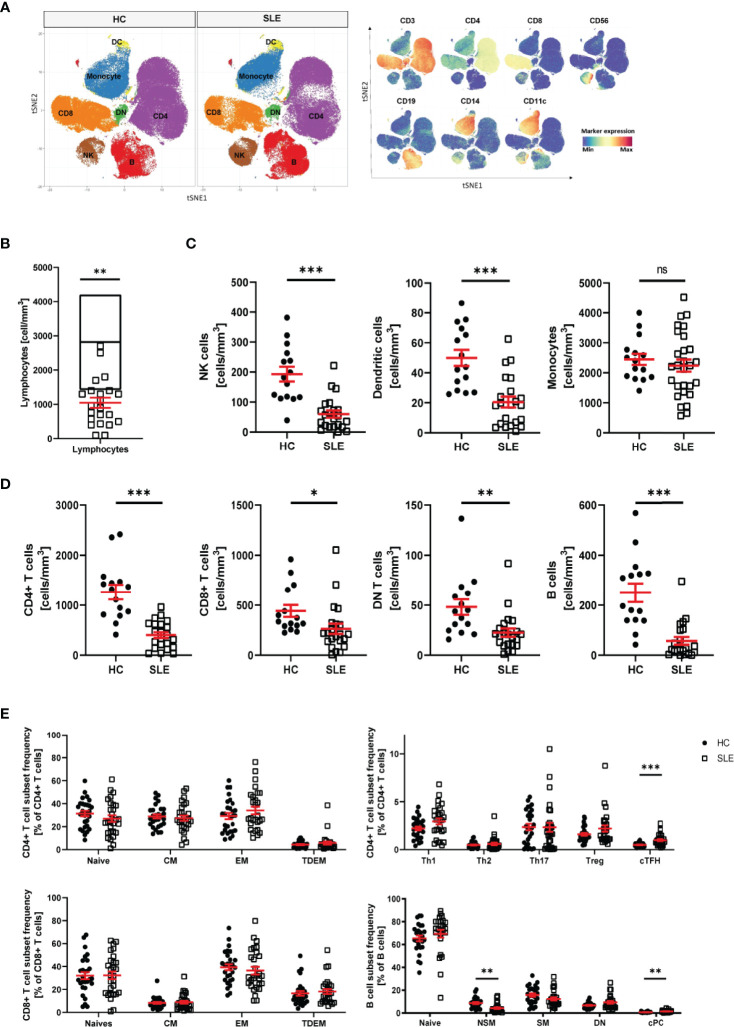
Distribution of PBMC in SLE patients. **(A)** T-stochastic neighboring embedding analysis of main PBMC populations on 3 representative SLE patients (with active disease) and mean expression of lineage markers (blue: low expression, red: high expression). **(B)** Lymphocyte abundance in SLE patients (n=22) compared to normal healthy range (represented as median with interquartile range of HC n=15, Student T-test, **p=0.007). **(C)** Abundance of innate immune cells in peripheral blood of SLE patients compared to HC (Welch’s T test, NK cells (HC n=15, SLE n=22, ***p<0.001), DC (HC n=15, SLE n=22, ***p<0.001), monocytes (HC=15, SLE n=28, ns p=0.46). **(D)** Abundance of adaptive immune cells in peripheral blood of SLE patients compared to HC (Welch’s T-test, CD4+ T cells (HC n=15, SLE n=22, ***p<0.001), CD8+ T cells (HC n=15, SLE n=22, **p=0.03), DN T cells (HC n=15, SLE n=22, **p=0.009), B cells (HC n=15, SLE n=21, ***p<0.001). **(E)** Frequency of CD4+ T, CD8+ T and B cell subpopulations in HC and SLE patients (HC n=28, SLE n=28, Welch’s T tests on log10 transformed data, cTFH p<0.001, NSM p=0.004, cPC Mann-Whitney test, p<0.001). Healthy controls (HC), Systemic lupus erythematosus patients (SLE), double negative (DN), natural killer (NK) cells, dendritic cells (DC), central memory (CM) cells, effector memory (EM) cells, terminally differentiated effector memory (TDEM) cells, T helper (Th) cell, T regulatory (Treg) cells, circulating T follicular helper (cTfh) cells, non switch memory (NSM) cells, switch memory (SM) cells, circulating plasma cells (cPC). Data represent mean ± SEM (*p = 0.02, **p = 0.02, ***p < 0.001, ns not significant).

The pathophysiology of systemic lupus erythematosus (SLE) is characterized by alterations of the innate and adaptive immune system. To identify an immune signature for SLE, we performed single cell mass cytometry analysis. We included markers for all major PBMC populations, markers of differentiation and markers of activation. First, we assessed the distribution of the main populations of PBMC in healthy controls (HC) and SLE patients: CD4+ T cells, CD8+ T cells, double negative (DN) T cells, B cells, natural killer (NK) cells, dendritic cells (DC) and monocytes (**Figure 1A** and **Supplementary Figure 1**). Consistent with previous studies (25, 26), we observed significant lymphopenia in SLE patients compared with HC (**Figure 1B**) and significant decrease in all lymphocyte subpopulations (including CD4+ T cells, CD8+ T cells, DN T cells, B cells, NK cells and DC) (**Figures 1C, D**), validating our technical approach. No difference was observed in abundance of monocytes in SLE patients compared to HC (**Figure 1C**). Interestingly, there was no association between lymphocyte count and disease severity or treatments (**Supplementary Figure 2**). We proceeded by analyzing the subpopulations of CD4+ T, CD8+ T and B cells (**Supplementary Figure 1**). The following populations were considered for CD4+ T cells: CD4+ T naïve, effector memory (EM), central memory (CM), terminally differentiated effector memory (TDEM), Th1, Th2, Th17, circulating T follicular helper cells (cTfh), regulatory T cells (Treg). T helper subsets were defined on the basis of cell surface chemokine receptor expression (**Supplementary Figure 1**). For CD8+ T cells, naïve, EM, CM and TDEM cells were included. Finally, for B cells, naïve, switch memory (SM), non-switch memory (NSM), double negative (DN) and circulating plasma cells (cPC) were included in the analysis (**Supplementary Figure 1**). We observed that the frequency of cTfh cells (CD45RO+CXCR5+) and cPC (CD27+CD38+) were significantly increased in patients with SLE (**Figure 1E**). The frequency of NSM B cells (CD27+IgD+) was significantly reduced in SLE patients. No other significant alterations in subset frequency were observed.

### Expression of SLAMF Receptors is Altered in Patients with SLE

**Figure 2:**
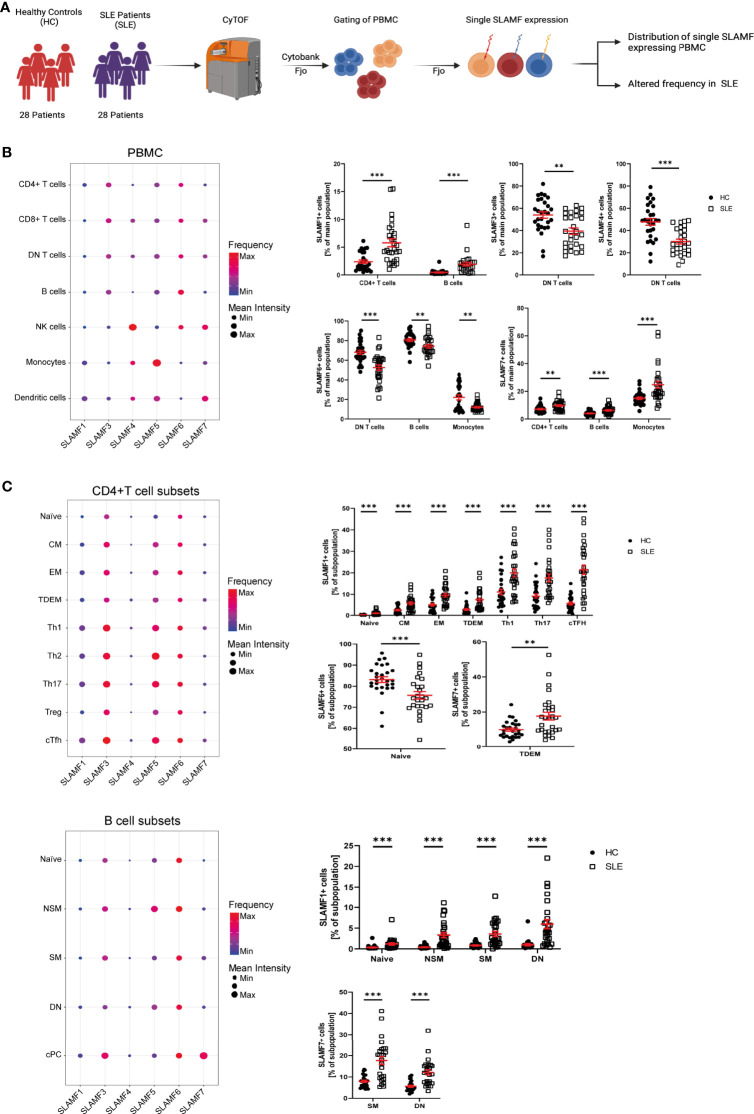
Single SLAMF expression in SLE. **(A)** Graphical abstract of technical approach **(B)** Dotplot of SLAMF expression in main PBMC of SLE patients (frequency and mean intensity, left) and presentation of significant differences in frequency of single SLAMF expressing PBMC between HC and SLE patients (n=28, Welch’s T tests on log10 transformed data, right). **(C)** Dotplot of SLAMF expression in CD4+ T (top) and B (bottom) cell subpopulations of SLE patients (frequency and mean intensity, left) and presentation of significant differences in frequency of single SLAMF expressing subpopulations between HC and SLE patients (n=28 for CD4+ T cells and n=26 for B cells, Welch’s T tests on log10 transformed data, right). DN T, double negative T cells; CM, central memory cells; EM, effector memory cells; TDEM, terminally differentiated effector memory cells; Th1, 2, 17, T helper type 1, 2, 17 cells; cTFH, circulating T follicular helper cells; NSM, non-switch memory cells; SM, switch memory cells; DN, double negative B cells; cPC, circulating plasma cells; Min, minimum mean intensity of marker expression; Max, maximum mean intensity of marker expression. Data represent mean ± SEM (**p= 0.02, ***p < 0.001).

Several studies indicate that SLAMF receptors play a role in the pathophysiology of SLE, as mentioned above (4). We hypothesize that SLAMF receptors expression defines an immune signature unique to SLE. To investigate this, we first examined the individual expression of each SLAMF receptor (SLAMF1, SLAMF3, SLAMF4, SLAMF5, SLAMF6, SLAMF7) on all main populations and subpopulation of PBMC from SLE patients included in cohort 1 (**Figure 2A**).

SLE patients showed a significant increase in the frequency of CD4+ T cells- and B cells-expressing SLAMF1, as well as CD4+ T cells-, B cells- and monocytes-expressing SLAMF7. Furthermore, there was a decrease in the frequency of DN T cells positive for SLAMF3 and SLAMF4, and of the percentage of DN T cells-, B cells- and monocytes-expressing SLAMF6 (**Figure 2B**). Next, we investigated SLAMF receptors expression on CD4+ T cell, CD8+ T cell and B cell subsets. We found that the percentage of SLAMF1-expressing cells was increased in all SLE CD4+ T cell subsets, including naïve T cells, CM, EM, TDEM, Th1, Th2, Th17, Treg and cTfh cells. Furthermore, the frequency of CD4+ TDEM-expressing SLAMF7 was significantly increased in SLE (**Figure 2C**). No significant alteration was observed in the expression of SLAMF receptors in SLE CD8+ T cell subsets (**Supplementary Figure 3**). Analysis of SLE B cell subsets indicated an increase in the frequency of naïve, NSM, SM and DN (CD27- IgD-) B cells-expressing SLAMF1. In addition, the frequencies of SM and DN B cells-expressing SLAMF7 were increased in SLE, whereas naïve B cells-expressing SLAMF6 were reduced in SLE patients compared to HC (**Figure 2C**).

### Expression of SLAMF Receptors is Linked to SLE Disease Activity

**Figure 3:**
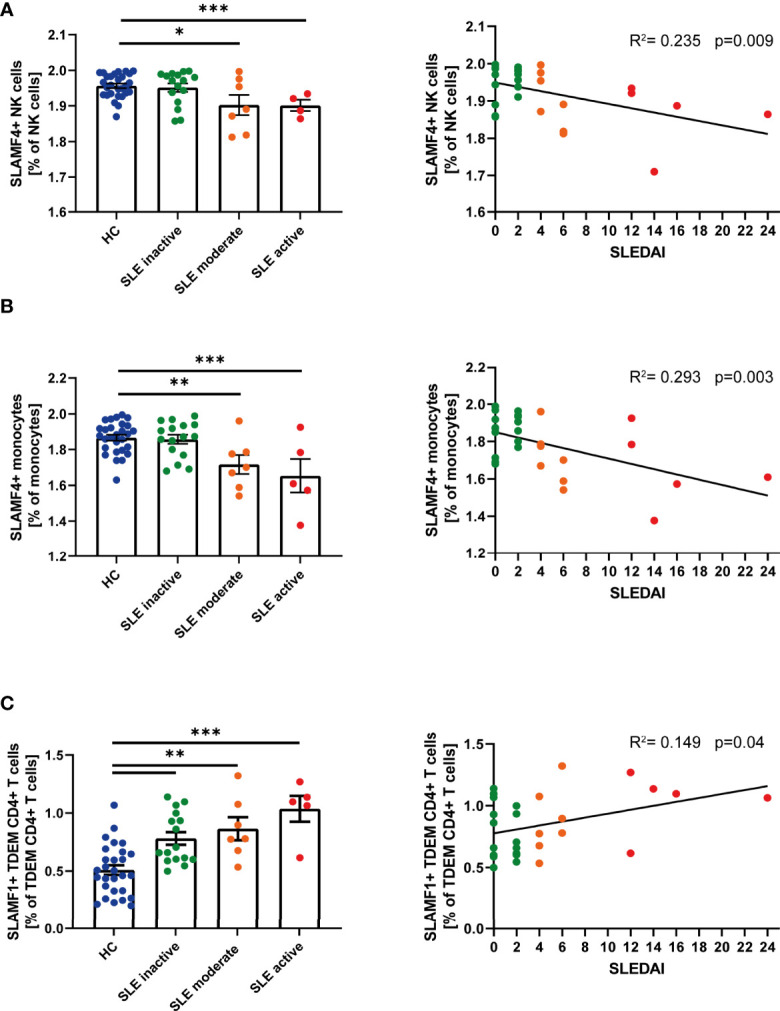
Single SLAMF link to disease activity. **(A)** Frequency of SLAMF4+ natural killer (NK) cells according to disease activity categories (one way ANOVA with Tukey’s multiple comparison test, left) and correlation between frequency of SLAMF4+ NK cells and SLEDAI (Pearson’s correlation, p=0.003, right). **(B)** Frequency of SLAMF4+ monocytes according to disease activity categories (one way ANOVA with Tukey’s multiple comparison test, left) and correlation between frequency of SLAMF4+ monocytes and SLEDAI (Pearson’s correlation, p<0.001, right). **(C)** Frequency of SLAMF1+ terminally differentiated effector memory CD4+ T (TDEM) cells according to disease activity categories (one way ANOVA, left) and correlation between frequency of SLAMF1+ TDEM and SLEDAI (Pearson’s correlation, p=0.04, right). Data presented as log10 transformed values,*p=0.02, **p=0.002, ***p<0.001.

Given the suggested relationship between SLAMF receptor expression and the pathophysiology of SLE, we questioned whether single SLAMF expression could serve as marker for disease activity. To answer this question, we first evaluated the individual expression of SLAMF on each population (**Supplementary Figure 4**) and subpopulation of PBMC. This analysis showed that the frequency of NK cells expressing SLAMF4 NK cells (**Figure 3A**) and of monocytes expressing SLAMF4 (**Figure 3B**) inversely correlated with disease activity. Furthermore, the percentage of TDEM CD4+ T expressing SLAMF1 positively correlated with SLE disease activity (**Figure 3C**).

### Identification of the Co-Expression of Multiple SLAMF Receptors at Single-Cell Level in SLE Patients

**Figure 4:**
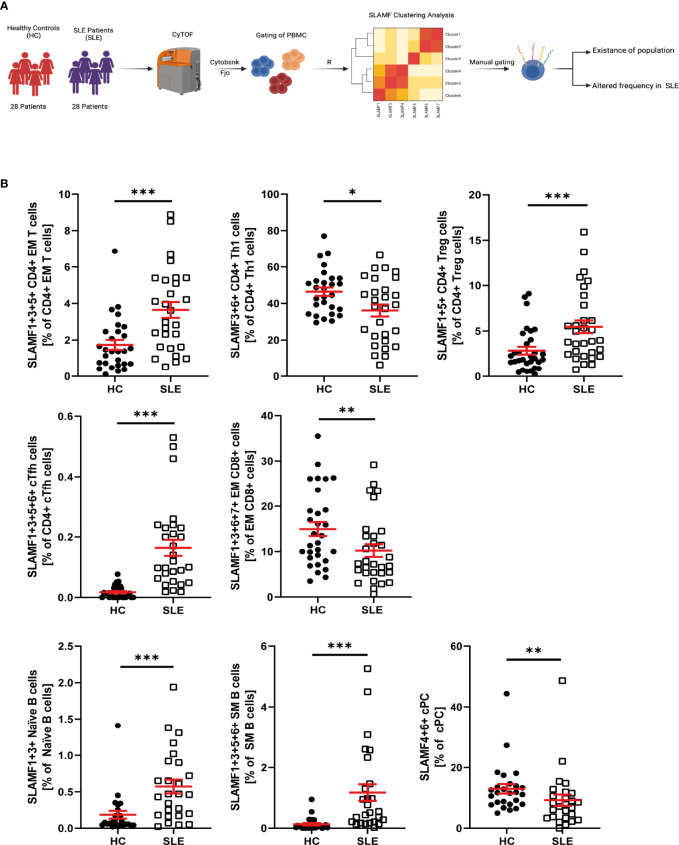
SLAMF co-expressing PBMC in HC and SLE patients. **(A)** Graphical abstract of technical approach. **(B)** Frequency of SLAMF co-expressing populations identified by consensus clustering (T test on log10 transformed data, Welch’s T test if normal, Mann-Whitney if not normal distributed). Data represent mean ± SEM (*p = 0.02, **p = 0.02, ***p < 0.001).

We evaluated the simultaneous expression of all SLAMF receptors at single-cell level (**Figure 4A**). To run this analysis, we performed unsupervised clustering analysis based on SLAMF expression on pre-gated major PBMC populations. This analysis was followed by unbiased clustering analysis of pre-gated subpopulations of CD4+ T cells, CD8+ T cells, B cells, NK cells, monocytes, and dendritic cells. Populations that were consistently discovered after applying sequential unbiased analysis were manually gated. Based on their relative cell abundance they may be of biological significance (**Supplementary Figure 6A**).

Accordingly, our analysis of CD4+ T identified the following cell subsets, which did not differ in their frequency between SLE and HC, as potentially relevant: naïve CD4+T cells and CM CD4+ T cells co-expressing SLAMF3 and SLAMF6, Th1 CD4+ T cells co-expressing SLAMF1, SLAMF3 and SLAMF6, Th2 CD4+ T cells co-expressing SLAMF5 and SLAMF6, Th17 CD4+ T cells co-expressing SLAMF3, SLAMF5 and SLAMF6 (**Supplementary Figure 5**). The presence of the following populations was confirmed after manual gating and their frequency was increased in SLE patients compared to HC: EM CD4+ T cells co-expressing SLAMF1, SLAMF3 and SLAMF5, Treg (CD127-CD25high) CD4+ T cells co-expressing SLAMF1 and SLAMF5, and cTFH CD4+ T cells co-expressing SLAMF1, SLAMF3, SLAMF5 and SLAMF6 (**Figure 4B**). Finally, Th1 CD4+ T cells co-expressing SLAMF3 and SLAMF6 were significantly decreased in SLE patients (**Figure 4B**). The frequency of these popualtions was not directly impacted by disease activity or treatments (**Supplementary Figure 7**)

The analysis of CD8+ T cells and subsets identified that EM CD8+ T cell co-expressing SLAMF1, SLAMF3, SLAMF6 and SLAMF7, were significantly decreased in SLE patients (**Figure 4B**). The frequency of these popualtions was not directly impacted by disease activity or treatments (**Supplementary Figure 7**)

Analysis of B cells and B cell subsets, identified one cell subset as potentially relevant, whose frequency was not significantly different between HC and SLE patients: naïve B cells co-expressing SLAMF3 and SLAMF6 (**Supplementary Figure 5**). Furthermore, naïve B cells co-expressing SLAMF1 and SLAMF3 and SM B cells co-expressing SLAMF1, SLAMF3, SLAMF5 and SLAMF6 were significantly increased in SLE, while cPC co-expressing SLAMF4 and SLAMF6 were reduced (**Figure 4B**). The frequency of these popualtions was not directly impacted by disease activity or treatments (**Supplementary Figure 7**)

From our analysis, we did not identify any SLAMF-based clusters in NK cells, DC and monocytes that exhibit altered frequency in patients with SLE compared to HC. However, CD16+PD1+ monocytes co-expressing SLAMF1, SLAMF5 and SLAMF7+ showed a tendency to be increased in patient with SLE. Furthermore, CD16high NK cells co-expressing SLAMF4, SLAMF6 and SLAMF7 are consistently identified by unbiased clustering analysis and their presence was confirmed by manual gating (**Supplementary Figure 5**).

### SLAMF Expression and Co-Expression Characterizes Patients With SLE Compared to Other Autoimmune Diseases

**Figure 5:**
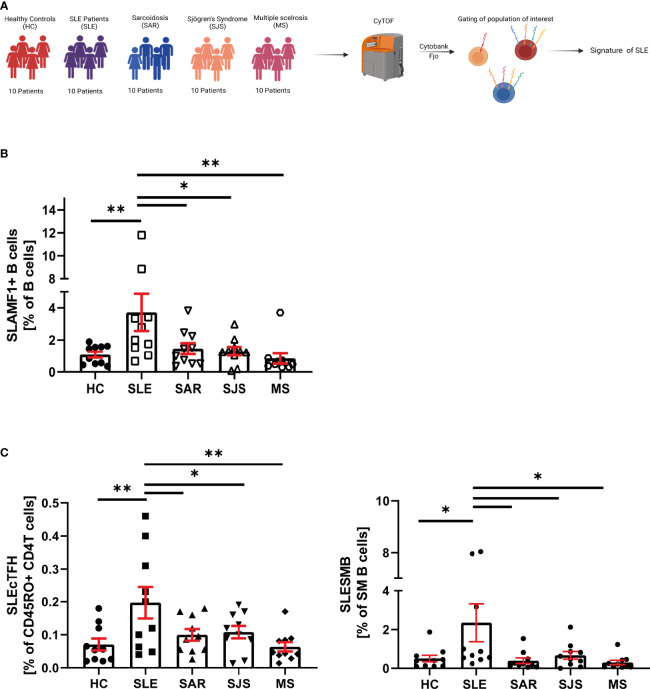
SLE specificity of SLAMF immune signature. **(A)** Graphical abstract of technical approach. **(B)** Frequency of SLAMF1+ B cells over B cells in cohort 2 (n=10 per group, one way ANOVA with Dunnett’s multiple comparison test on log10 transformed data, *p=0.02, **p=0.002, ***p<0.001). **(C)** Frequency of circulating T follicular helper cells expressing SLAMF1+3+5+6 over memory CD4+ T cells (left) and switch memory B cells expressing SLAMF1+3+5+6+ over SM B cells (right) in cohort 2 (n= 10 per group, one way ANOVA with Dunnett’s multiple comparison test on log10 transformed data, *p=0.02, **p=0.002).

In order to identify an immune signature specific to SLE, we considered a second cohort of patients (cohort 2), which included patients with SLE, HC and patients with the following autoimmune diseases: sarcoidosis (SAR), Sjögren’s syndrome (SJS) and multiple sclerosis (MS) (**Figure 5A**).

In patients included in the cohort 2, we examined SLAMF-based cell populations that were identified in the cohort 1. We first focused our analysis on single SLAMF receptor expression. We observed that, among all the populations of interest identified in the cohort 1, only B cells-expressing SLAMF1 (identified as SLEB1) were significantly increased in SLE compared to healthy and autoimmune diseases controls (**Figure 5B**). Then, we examined the frequencies of population defined by the co-expression of multiple SLAMF receptors as characteristics of SLE in cohort 1. This analysis showed that two populations are significantly increased in SLE patients compared to healthy and autoimmune diseases controls (**Figure 5C**): SM B cells co-expressing SLAMF1, SLAMF3, SLAMF5 and SLAMF6 (identified as SLESMB) and cTfh CD4+ T cells co-expressing SLAMF1, SLAMF3, SLAMF5 and SLAMF6 receptors (identified as SLEcTFH).

### Identification of an Immune Signature for SLE Based on the Expression of SLAMF Receptors by PBMC

**Figure 6:**
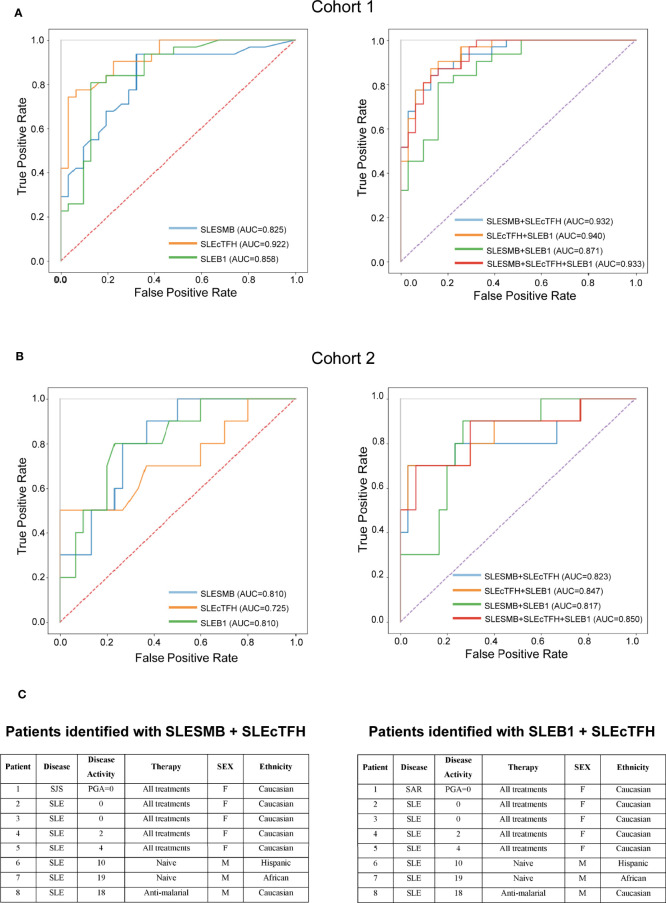
Predictive value of SLAMF expressing populations for SLE. **(A)** ROC curves of SLESMB, SLEcTFH and SLEB1 and of their combinations in cohort1. **(B)** ROC curves of SLESMB, SLEcTFH and SLEB1 and of their combinations in cohort 2. **(C)** Samples of cohort 2 identified by combining SLESMB-SLEcTFH and SLEB1-cTFH.

Overall, analysis of single-expression and co-expression of SLAMF receptors in PBMC identified three subsets of cells with altered frequencies in SLE compared to healthy and autoimmune controls. We investigated the potential of each of these populations, taken individually or in combination, to distinguish SLE patients from healthy individuals and patients with other autoimmune diseases. The populations of interest were present in healthy and autoimmune disease individuals. Therefore, we used their frequencies and evaluated them as continuous variables. To compare SLE and HC, the frequency of each cell subset was normalized (min-to-max normalization). Then, we determined which cell populations taken separately was the best marker to differentiate SLE patients from healthy controls. ROC curves showed that the measurement of the SLEcTFH population was the best individual marker (AUC = 0.92) to distinguish SLE from HC (**Figure 6A**). Then, we combined the normalized values of the different populations to determine which combination best discriminates SLE from HC. We observed that the measurements of SLESMB together with SLEcTFH increases the performance from SLESMB AUC = 0.83, SLEcTFH AUC = 0.92 to AUC = 0.94 (**Figure 6A**).

Secondly, we examined which cell population (normalized frequencies) best distinguishes SLE from other autoimmune diseases using subjects included in the cohort 2. We observed that the single measurement of SLEB1 and SLESMB better discriminates SLE from autoimmune controls compared to SLEcTFH (SLEB1/SLESMB AUC = 0.81 vs. SLEcTFH AUC = 0.72, **Figure 6B** and **Supplementary Figure 8**). Furthermore, the combined measurements of SLEB1 and SLEcTFH taken together was the best to differentiate SLE from autoimmune diseases (AUC = 0.847, **Figure 6B** and **Supplementary Figure 8**). We then calculated the ideal cut-off to distinguish SLE from autoimmune diseases controls in cohort 2. Using the Youden index, we determined that individuals with a score greater than 0.282 (for both the SLEB1-SLEcTFH and SLESMB-SLEcTFH combinations) can be diagnosed as SLE. Furthermore, we evaluated whether the measurements of these cell populations were associated with specific clinical characteristics of SLE (cohort 1). Our data suggest that the measurement of SLEcTFH and the combined measurement of SLESMB-SLEcTFH discriminate patients with arthritis (**Supplementary Figure 9**). Nevertheless, a larger sample size, presenting varying organ involvement, is necessary to confirm these associations. Overall, our data indicate that the combination of SLEB1 and SLEcTFH measurements or the combination of SLESMB and SLEcTFH, both correctly diagnosed 90% of SLE samples (**Figure 6C** and **Supplementary Table 1C**). These results show that the expression of SLAMF receptors by PBMC can represent a powerful diagnostic tool for SLE.

## Discussion

To identify a SLE specific immune signature, based on SLAMF receptors expression, we used single-cell mass cytometry to perform an in-depth analysis of the main PBMC populations. Compared to previous studies, which focused on one or on a few SLAMF receptors at a time, this technique allowed the simultaneous examination of all different SLAMF molecules present on peripheral immune cells.

Our data identified that the frequency of SLAMF1+ B cells (SLEB1) is significantly increased in SLE patients compared to all controls (healthy and autoimmune). Moreover, consensus clustering analysis identified alteration in the frequencies of several populations co-expressing SLAMF receptors in SLE patients compared to healthy controls. The frequencies of SMB cells and cTFH cells co-expressing SLAMF1, SLAMF3, SLAMF5 and SLAMF6 (identified as SLESMB and SLEcTFH, respectively) were significantly increased in SLE compared to all controls (healthy and autoimmune). We showed that the increased frequency of SLEB1, SLESMB and SLEcTFH is sufficient to discriminate SLE patients from sarcoidosis, Sjögren’s syndrome and multiple sclerosis patients. Furthermore, the combined measurements of SLEB1-SLEcTFH or SLESMB-SLEcTFH increased the accuracy of discrimination. Indeed, 90% of the individuals identified with this approach were diagnosed with SLE. Interestingly, SLE patients identified by this approach have varying clinical characteristic, disease activity and are treated heterogeneously. This suggests that the analysis of these cell populations identifies patients with SLE independently of clinical disease presentation and treatments. In the majority of the cases, these differences do not prevent their identification using the above-mentioned markers. Our data identified three cell subsets that correlated with disease activity: the frequency of SLAMF4+ NK cells and SLAMF4+ monocytes was inversely correlated with SLEDAI, while the frequency of SLAMF1+ TDEM CD4+ T cells was directly correlated with disease activity. Overall, our data show that the expression of SLAMF receptors defines an immune signature that is specific to SLE. Moreover, our data further suggest a role of SLAMF receptors in the pathophysiology of SLE, as previously shown in human and murine models (8–10, 14, 18, 19, 27, 28).

The expression of single-SLAMF receptors by PBMC population and subpopulations closely matched previously published results (17). There are minor differences between the two studies, which mainly concern the expression of SLAMF6 and SLAMF7. These distinctions may be related to variations in the composition of the cohorts and to differences in the technique used (flow vs. mass cytometry). From this point of view, mass cytometry can be less sensitive to detect molecules with low level of expression compared to flow cytometry (29, 30).

SLAMF1 has been shown to be increased in T and B cells of SLE patients upon activation (17, 27). In addition, this receptor is implicated in B cell proliferation, differentiation and Ig production (31). Targeting SLAMF1 has been proposed as a therapeutic target for SLE since anti-SLAMF1 monoclonal antibody can reduce the T-B interaction, B cell production of IL6 and B cell differentiation into plasma cells (8).

To our knowledge, no study to date has examined the co-expression of SLAMF receptors in SLE PBMC. Our analysis shows that two cell populations, defined on the basis of the co-expression of SLAMF receptors, are altered in frequency in SLE patients compared to healthy and autoimmune controls. These populations are SLESMB and SLEcTFH cells, which both co-express SLAMF1, SLAMF3, SLAMF5 and SLAMF6. Since SLAMF receptors act as self-ligands and are expressed on both populations, they likely play a role in the cellular interaction of SLESMB and SLEcTFH cells. Functional studies will be essential to deeper understand the role of these cell populations in SLE patients. From this point of view, a study on mice with disrupted SLAMF1, SLAMF5 and SLAMF6 genes showed that these receptors synergistically contribute to humoral immunity control (32). Indeed, SLAMF1-SLAMF5-SLAMF6- mice exhibited an increased T-dependent and T-independent production of antibodies compared to the wild type. Moreover, SLAMF3 deficient mice develop autoimmune features, including the expansion of Tfh cells and germinal center B cells and the production of autoantibodies, suggesting a role in the regulation of humoral immunity (7). Although no studies has evaluated the absence of all four receptors at the same time, these murine models suggest that SLAMF1, SLAMF3, SLAMF5 and SLAMF6 may be responsible for the fine-tuning of regulation of humoral immunity.

SLE is a very heterogeneous disease with great variability in susceptibility factors and symptoms. For this reason, there is often a significant delay between the first symptoms and the definitive SLE diagnosis. This can delay adequate medical management and lead to permanent organ damage. Accordingly, discovering biomarkers that are both specific and sensitive enough to identify all patients suffering from SLE is an important goal to achieve. The markers we propose here can identify the vast majority of SLE patients, despite significant clinical presentation heterogeneity. However, cytometers that allow simultaneous analysis of the large number of cell surface markers needed for this approach might not be readily available to most diagnostic laboratories.

The major limitations of this research are the relatively small size of the cohorts studied and that the patients included are almost exclusively Caucasian. Further studies are needed to confirm the validity of our findings in other ethnic populations. In addition, examining a larger cohort may better define the optimal cutoff for our biomarkers and increase the specificity of the test. Furthermore, a cohort including more patients with active disease is warranted to confirm the findings on the correlation of SLAMF4+ NK cells, SLAMF4+ monocytes and TDEM CD4+ SLAMF1+ with disease activity. Moreover, patients with active organ involvement should be included to evaluate if these cell subsets could be used to predict organ involvement.

In conclusion, this study identified an immune signature based on the expression of SLAMF receptors by PBMC, which is specific for SLE and may represent a biomarker to identify the disease and its severity.  

## Data Availability Statement

The raw data supporting the conclusions of this article will be made available by the authors, without undue reservation.

## Ethics Statement

The studies involving human participants were reviewed and approved by SwissEthics 2017-01434 and 2018-01622. The patients/participants provided their written informed consent to participate in this study.

## Author Contributions

DC: study design. MH and FB: conducting experiments. MH, MS, and MM: data analysis. DC, CR, AH, AM, RDP: recruitment of patients and controls. CF: responsible for CyTOF facility. DC, MH, and NF: writing and editing of manuscript. All authors reviewed the manuscript. All authors contributed to the article and approved the submitted version.

## Funding

This study received funding from the Swiss National Science Foundation (Ambizione PZ00P3_173950 to DC) and a grant from the Novartis Foundation for Medical-Biological Research (to DC). The funder was not involved in the study design, collection, analysis, interpretation of data, the writing of this article, or the decision to submit it for publication.

## Conflict of Interest

The authors declare that the research was conducted in the absence of any commercial or financial relationships that could be construed as a potential conflict of interest.

## Publisher’s Note

All claims expressed in this article are solely those of the authors and do not necessarily represent those of their affiliated organizations, or those of the publisher, the editors and the reviewers. Any product that may be evaluated in this article, or claim that may be made by its manufacturer, is not guaranteed or endorsed by the publisher.
