# Supplementary material for: SLAMF Receptor Expression Identifies an Immune Signature That Characterizes Systemic Lupus Erythematosus
Source: Front Immunol. 2022 May 6;13:843059. doi: 10.3389/fimmu.2022.843059 (PMC9120573; doi:10.3389/fimmu.2022.843059)
Supplement: Supplementary file 1 [file DataSheet_1.pdf]

Supplementary Material

Supplementary Table 1. Patient information

A. Cohort 1 Patient information

Information	HC (n=28)	SLE (n=28)
Age, mean (SD)	42 (14)	42 (14)
Gender (% female)	82%	82%
Ethnicity (% Caucasian)	89%	89%
Disease Activity (n)<br>Active SLE<br>Moderate SLE<br>Inactive SLE	--	 5<br>7<br>16
Therapy<br>Naïve<br>Antimalarials only<br>Immunosuppressants	--	6<br>8<br>14
Clinical Feature (n)<br>Fever<br>Arthritis<br>Myositis<br>Mucocutaneous<br>Vasculitis<br>Serositis<br>Neurological<br>Hematological<br>Renal<br>Serological	--	1<br>3<br>0<br>5<br>0<br>0<br>0<br>2<br>6<br>18

HC: healthy controls, SLE: Systemic lupus erythematosus, Disease activity (Active SLE: SLEDAI>10, moderate SLE: SLEDAI 3-10, inactive SLE: SLEDAI<3), Therapy: naïve was considered patients with no rituximab ever and no immunomodulatory treatment during month prior to sampling; antimalarials only (Hydroxychloroquine, average daily dose (263pm, SD=106), no cytotoxic drug or biologic agent), Immunosuppressants (corticosteroids (oral prednisone, n=11, average daily dose 8.3mg, SD=5.9), hydroxychloroquine (n=10, average daily dose 274pm,

SD=85), azathioprine (n=5, average daily dose 75, SD=35), mycophenolate mofetil (n=5, average daily dose 1000, SD=612).

SD: Standard Deviation.

B. Cohort 2 Patient information

Information	HC (n=10)	SLE (n=10)	SAR (n=10)	SJS (n=10)	MS (n=10)
Age, mean (SD)	36 (17)	37 (17)	55 (12)	50 (18)	38 (8)
Gender (% female)	80%	70%	40%	100%	60%
Ethnicity (% Caucasian)	100%	80%	100%	90%	--
Disease Activity (%)	--				
High		30%	30%	40%	--
Moderate		20%	40%	20%	40%
Low		50%	30%	40%	60%
Therapy (% naïve)	--	20%	50%	50%	100%

HC: healthy controls, SLE: Systemic lupus erythematosus, SAR: sarcoidosis, SJS: Sjögren's Syndrome, MS: multiple sclerosis, PGA: Physician Global Assessment, EDSS: Expanded Disability Status Scale, SD: standard deviation. Disease activity per disease is categorized as follows: SLE SLEDAI (high<10, medium 4-10, low<3), SAR and SJS PGA (high=2, medium=1, low=0), MS EDSS (severe disability>3, moderate disability 2.5-3, 0-2 low disability). For therapy naïve patients with no rituximab and no immunomodulatory drugs in the month proceeding sampling were considered.

C. Clinical Features of identified patients

Patient	Mucocutaneous	Arthritis	Myositis	Serositis	Renal	Hematologic	Serologic
SLE 029	-	-	-	-	-	-	-
SLE 030	-	-	-	-	-	-	-
SLE 031	-	-	-	-	-	-	Yes
SLE 040	-	-	-	-	-	-	Yes
SLE 065	Yes	Yes	-	-	-	-	Yes
SLE 077	Yes	Yes	Yes	Yes	-	Yes	Yes
SLE 078	Yes	-	-	-	Yes	-	Yes

Fever, vasculitis and neurological manifestations were not observed among the patients.
SLE: Systemic lupus erythematosus, Mucocutaneous: patient had rash/mucosal ulcers/alopecia, Serositis: patient had pleurisy/ pericarditis, Renal: patient had urinary cast/hematuria/pyuria/proteinuria, Hematologic: patient had thrombocytopenia/leukopenia, Serologic: patient had low C3/low C4/ anti-dsDNA antibodies.

Supplementary Table 2. Antibody list

A. Barcoding Cohort 1

Mass Cytometry Antibody	Format	Clone	Company
CD45	89 Y	HI30	Fludigm
CD45	148 Nd	HI30	Conju-Biolegend
CD45	166 Er	HI30	Conju-Biolegend
CD45	198 Pt	HI30	Conju-Biolegend

B. Barcoding Cohort 2

Mass Cytometry Antibody	Format	Clone	Company
CD45	89 Y	HI30	Fludigm
CD45	194 Pt	HI30	Conju-Biolegend
CD45	195 Pt	HI30	Conju-Biolegend
CD45	196 Pt	HI30	Conju-Biolegend
CD45	198 Pt	HI30	Conju-Biolegend

C. Extracellular phenotyping Panel

Mass Cytometry Antibody	Format	Clone	Company
Live/Dead	103Rh	-	Fludigm
CD8	113 In	RPA-T8	Biolegend
CD4	115 In	RPA-T4	Biolegend
CD196/CCR6	141 Pr	11A9	Fludigm
CD19	142 Nd	HIB19	Fludigm
CD352 / SLAM 6	143 Nd	NT-7	Fludigm
CD38	144 Nd	HIT2	Biolegend
CD127	145 Nd	A019D5	Biolegend
IgD	146 Nd	IA6-2	BD bioscience
CD7	147 Sm	CD7-6B7	Fludigm
CCR4	149 Sm	205410	Fludigm
CD3	150 Nd	UCH-T1	BD bioscience
CD123	151 Eu	6H6	Fludigm
PD-1	151 Eu	EH12.2H7	Biolegend
CD21	152 Sm	BL13	Fludigm
CD45RA	153 Eu	HI100	BD bioscience
CD84 / SLAM 5	154 Sm	CD84.1.21	Fludigm
CD27	155 Gd	L128	Fludigm
CD319/SLAMF 7	156 Gd	162.1	Biolegend
CXCR3	158 Gd	1C6/CXCR3	BD bioscience
CCR7	159 Tb	G043H7	Biolegend
CD14	160 Gd	M5E2	Fludigm
CD150 / SLAM 1	161 Dy	A12(7D4)	Biolegend
CD11c	162 Dy	clone 3.9	Fludigm
CRTh2 (Fluidigm)	163 Dy	BM16	Fludigm
CD48 / SLAM 2	164 Dy	BJ40	Biolegend
CD45RO	165 Ho	UCHL1	Fludigm
CXCR5	167 Er	RF8B2	Biolegend
ICOS	168 Er	C398.4A	Biolegend
CD25	169 Tm	2A3	Fludigm
TCR va24-Ja18 (6B11)	170 Er	Witek	Fludigm
CD20	171Yb	2H7	Fludigm
TCRαβ	172 Yb	IP26	Biolegend
CD353/ SLAM8	172 Yb	REA394	Conju-Miltenyi
HLA-DR	173 Yb	L243	Fludigm
CD229 / SLAM 3	174 Yb	HLy9.1.25	Fludigm
CD244 / SLAM 4	175Lu	C1.7	Biolegend
CD56	176Yb	R19-760	Fludigm
CD57 (CHUV)	194 Pt	NK1	Conju-BD bioscience
CD16	209Bi	3G8	Fludigm

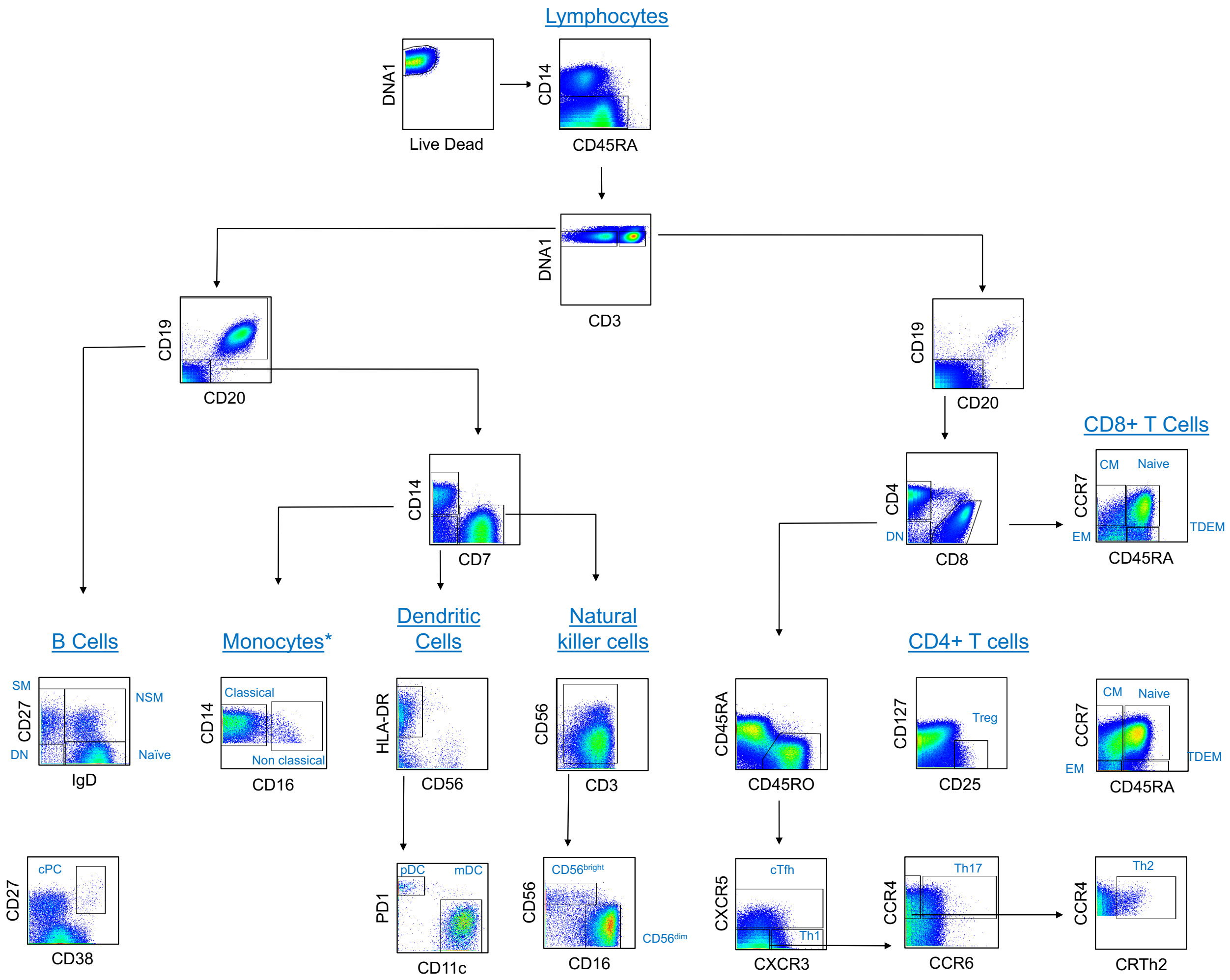

Supplementary Figure 1. Gating Strategy. Following debarcoding and removal of doublets, cells were gated as shown in this representative example. NSM: non-switch memory, SM: switch memory, DN: double negative, cPC: circulating plasma cells, pDC: plasmacytoid dendritic cells, mDC: myeloid dendritic cells, cTfh: circulating T follicular helper, Th1/2/17: T helper type 1/2/17, Treg: regulatory T, CM: central memory, EM: effector memory, TDEM: terminally differentiated effector memory.

A

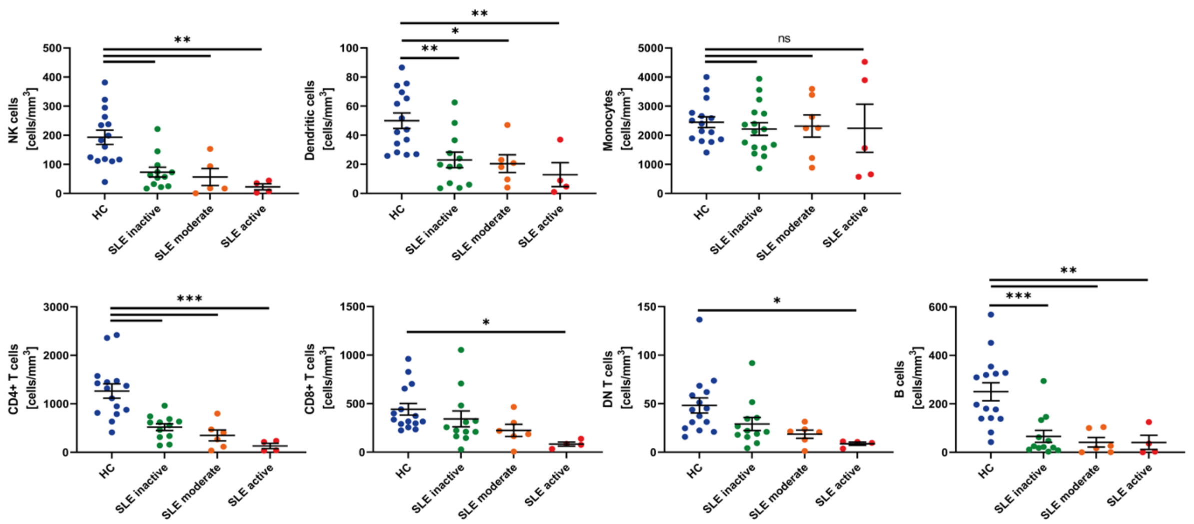

B

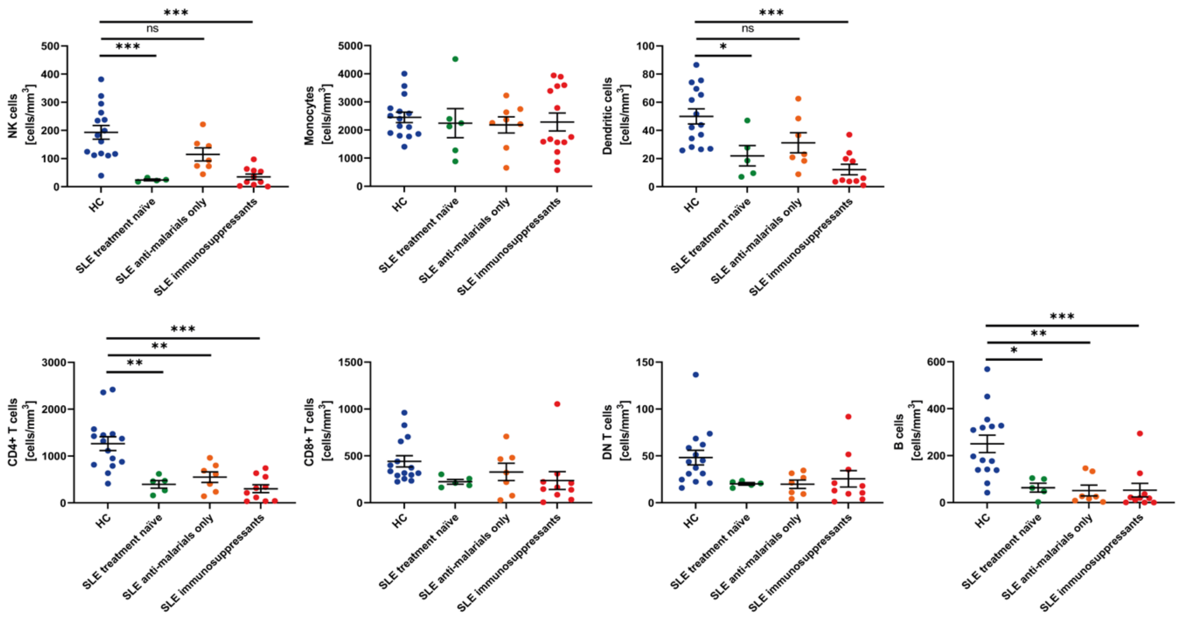

Supplementary Figure 2. PBMC abundance according to disease activity and treatments.
(A) Abundance of innate immune cells (top row) and adaptive immune cells (bottom row) according to disease activity (HC n=15, inactive SLE n=12, moderate SLE n=6, active SLE n=4, One way ANOVA, *p=0.02, **p=0.002, ***p<0.001). (B) Abundance of innate immune cells (top row) and adaptive immune cells (bottom row) according to treatment (HC n=15, SLE treatment naïve n=5, SLE anti-malarials only n=7, SLE immunosuppressants (see Supplementary Table 1A) n=10, One way ANOVA, *p=0.02, **p=0.002, ***p<0.001).

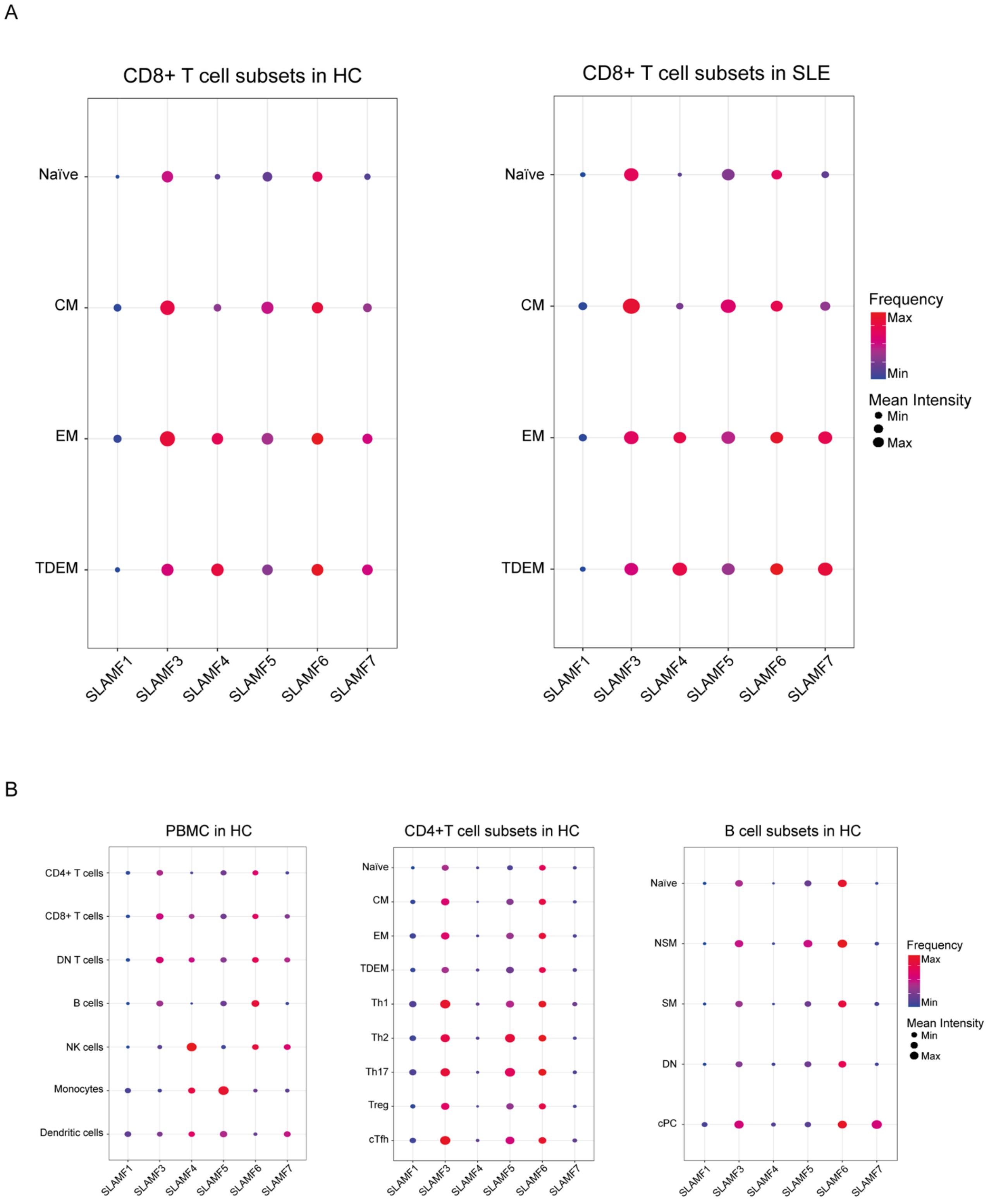

Supplementary Figure 3. SLAMF expression in SLE and in HC. (A) Dotplot of SLAMF expression in CD8+ T cell subpopulations in HC (n=28, left) and SLE patients (n=28, right) showing frequency and mean intensity. (B) Dotplots of SLAMF expression in PBMC (left), CD4+ T cell subsets (center) and B cell subsets (right) in healthy controls, showing frequency and mean intensity (n=28).

A

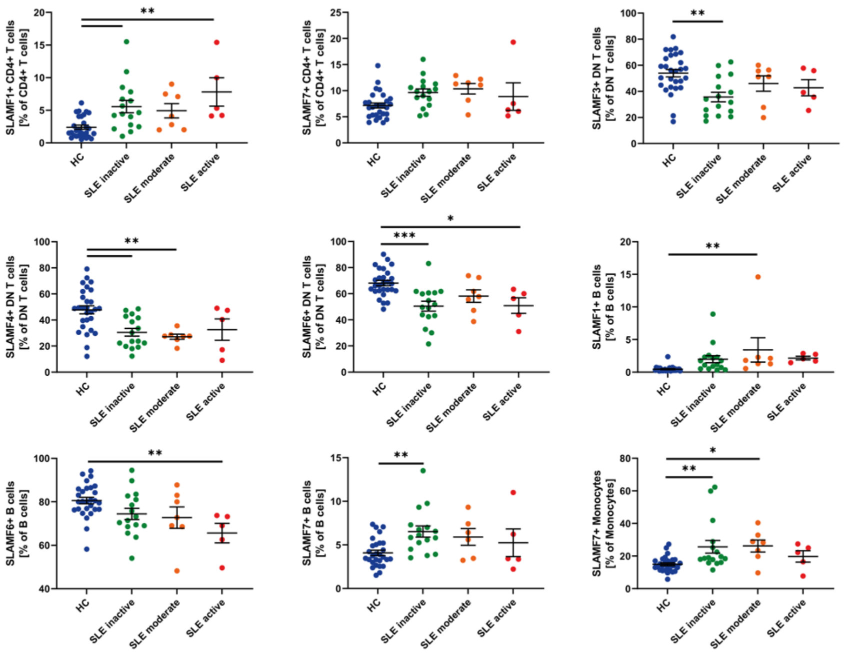

B

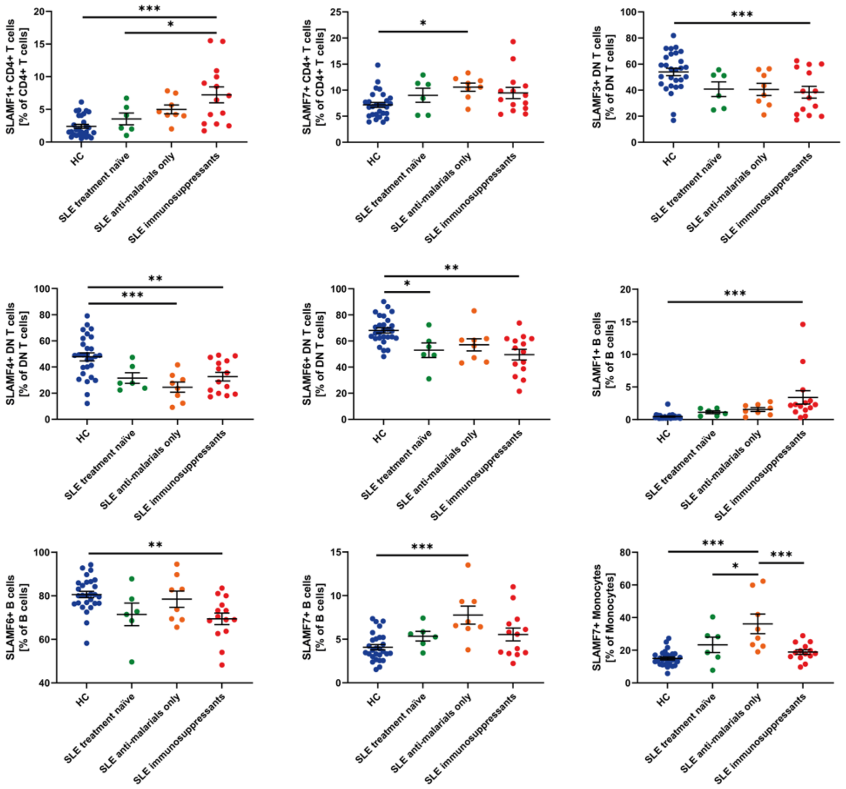

Supplementary Figure 4. Single SLAMF expressing PBMC frequency according to disease activity and treatments. (A) Frequency of single SLAMF expressing PBMC according to disease activity (HC n=28, inactive SLE n=16, moderate SLE n=7, active SLE n=5, One way ANOVA, *p=0.02, **p=0.002, ***p<0.001). (B) Frequency of single SLAMF expressing PBMC according to treatment (HC n=28, SLE treatment naïve n=6, SLE anti-malarials only n=8, SLE all else n=14, One way ANOVA, *p=0.02, **p=0.002, ***p<0.001).

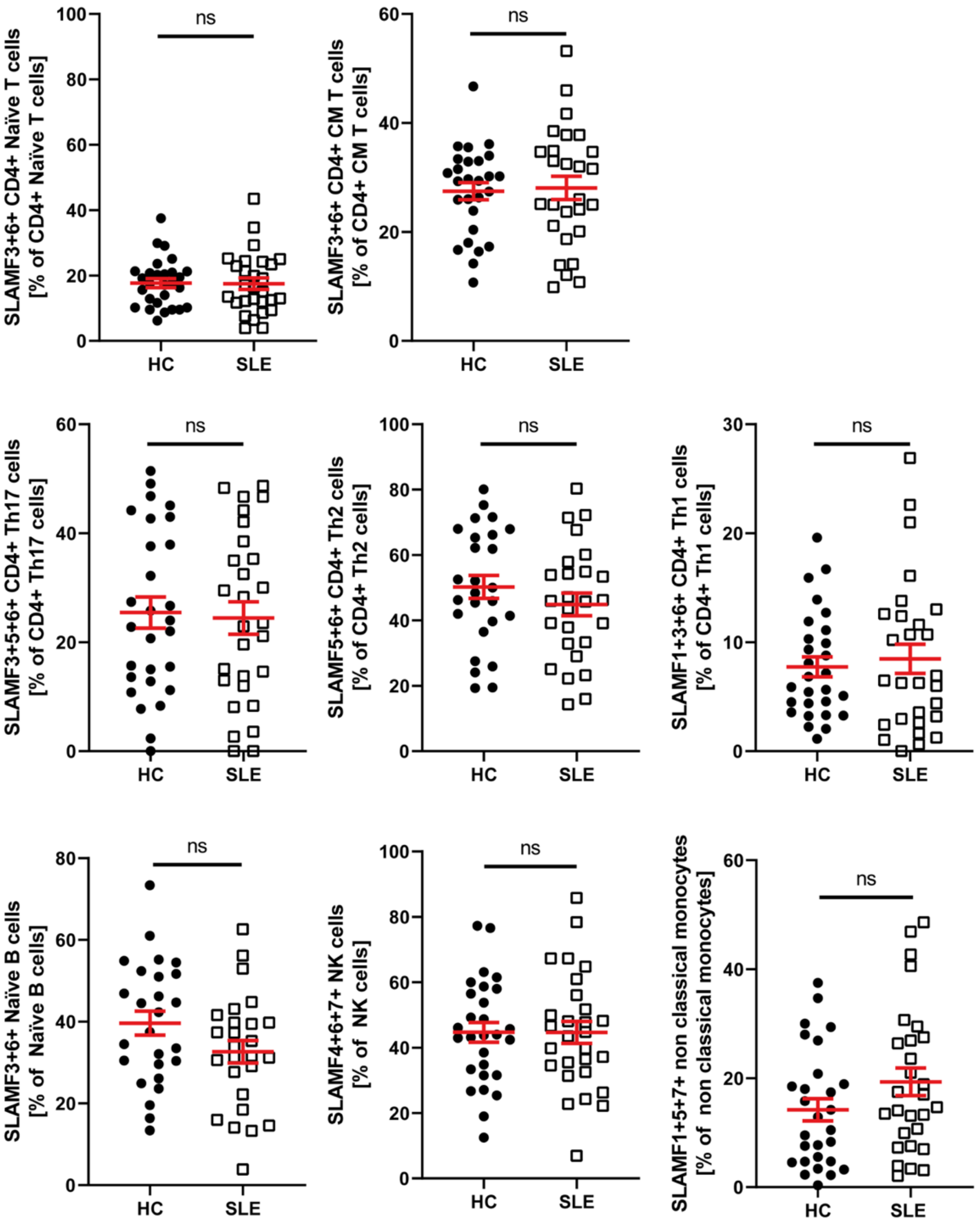

Supplementary Figure 5. Newly identified SLAMF co-expressing cell subsets. Frequency of SLAMF co-expressing populations identified by consensus clustering of cohort 1 and confirmed by manual gating, but not presenting significant alterations in SLE patients compared to HC (n=28, Welch T test on log10 transformed data).

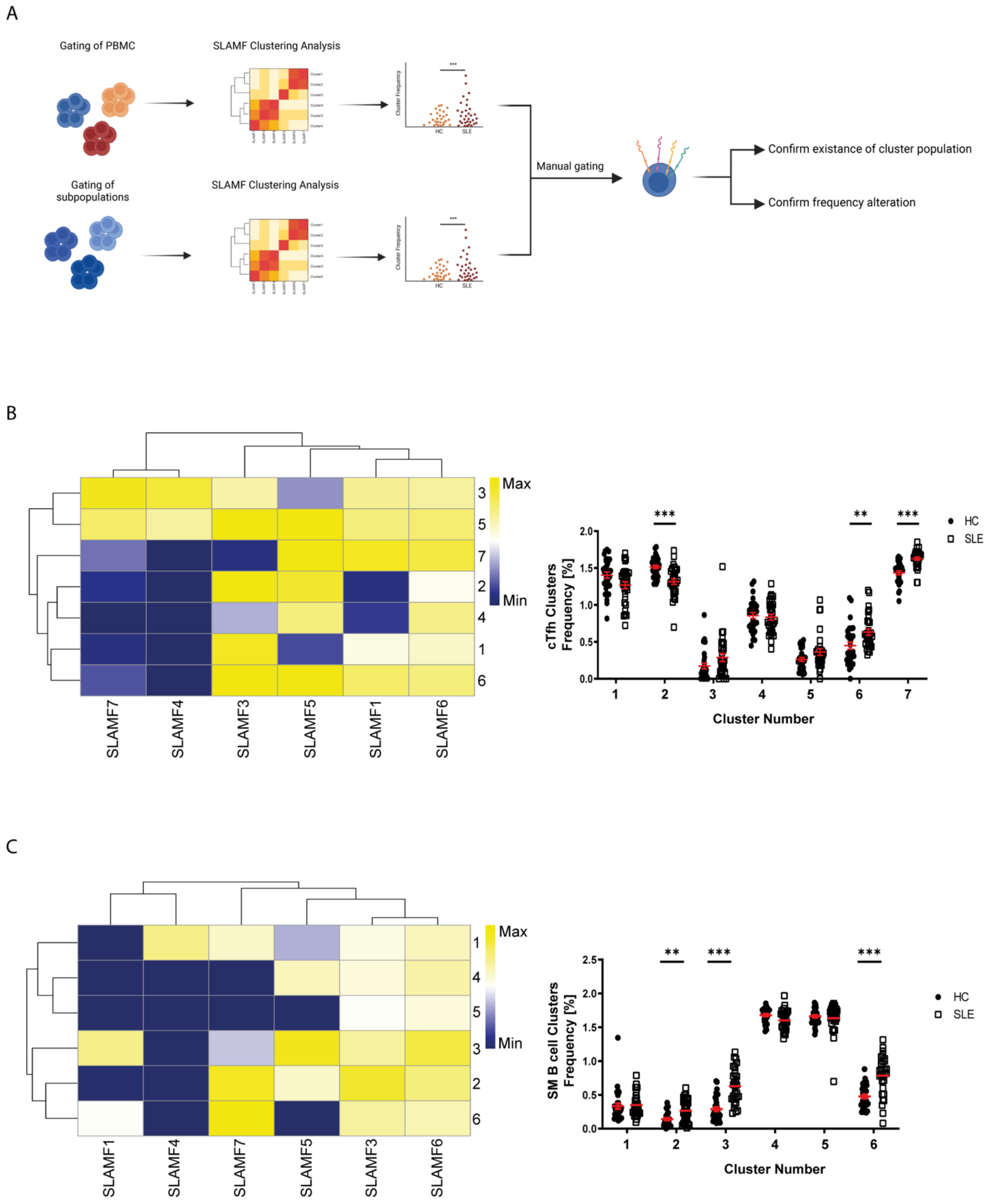

Supplementary Figure 6. Consensus clustering analysis identifies populations of interest. (A) Graphical abstract of technical approach. (B) Heatmap of clustering analysis of cTfh cells (left) and frequency of clusters in HC and SLE patients (n=28, Welch T test on log10 transformed data, right). (C) Heatmap of clustering analysis of switch memory B cells (left) and frequency of clusters in HC and SLE patients (n=26, Welch T test on log10 transformed data, right).

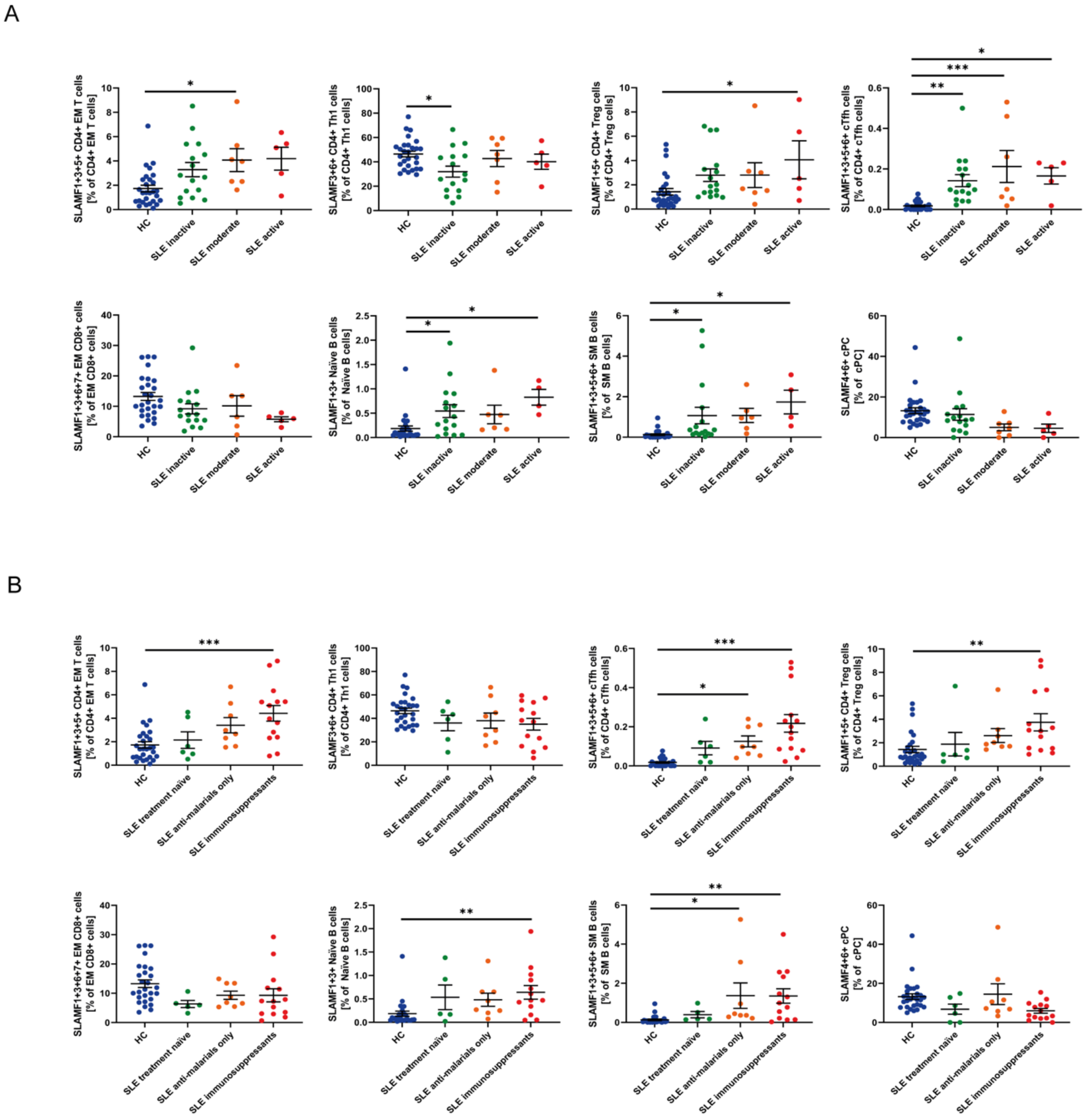

Supplementary Figure 7. Frequency of cluster populations according to disease activity and treatments. (A) Frequency of cluster populations according to disease activity (HC n=28, inactive SLE n=16, moderate SLE n=7, active SLE n=5, One way ANOVA, *p=0.02, **p=0.002, ***p<0.001). (B) Frequency of cluster populations according to treatment (HC n=28, SLE treatment naïve n=6, SLE anti-malarials only n=8, SLE immunosuppressants n=14, One way ANOVA, *p=0.02, **p=0.002, ***p<0.001).

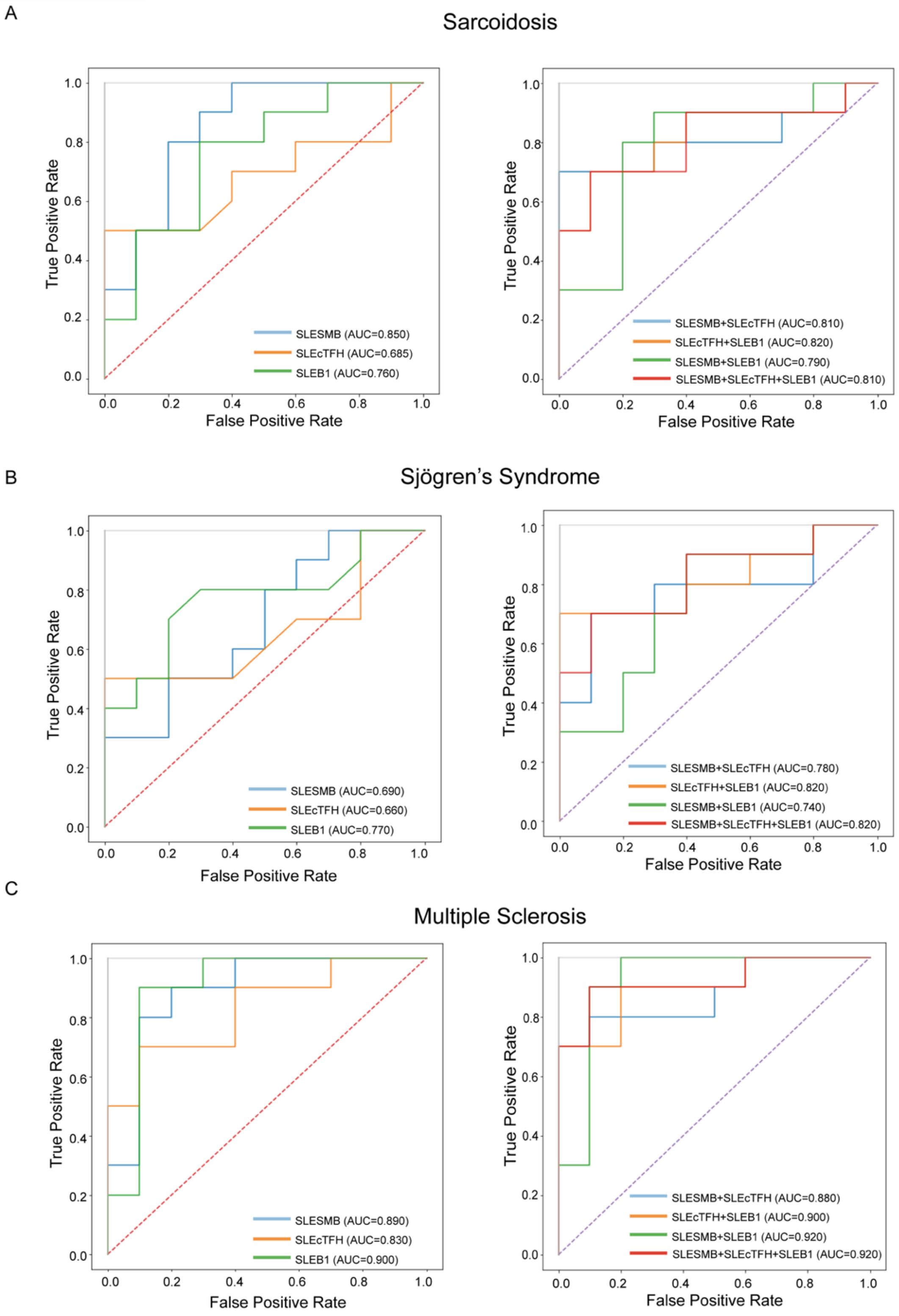

Supplementary Figure 8. ROC curve analysis for SLE against autoimmune diseases. (A) ROC curve analysis for SLESMB, SLEcTFH, SLEB1 in SLE versus sarcoidosis (left) and for the combined measurements of SLESMB, SLEcTFH and SLEB1 (right). (B) ROC curve analysis for SLESMB, SLEcTFH, SLEB1 in SLE versus Sjögren's syndrome (left) and for the combined measurements of SLESMB, SLEcTFH and SLEB1 (right). (C) ROC curve analysis for SLESMB, SLEcTFH, SLEB1 in SLE versus multiple sclerosis (left) and for the combined measurements of SLESMB, SLEcTFH and SLEB1 (right).

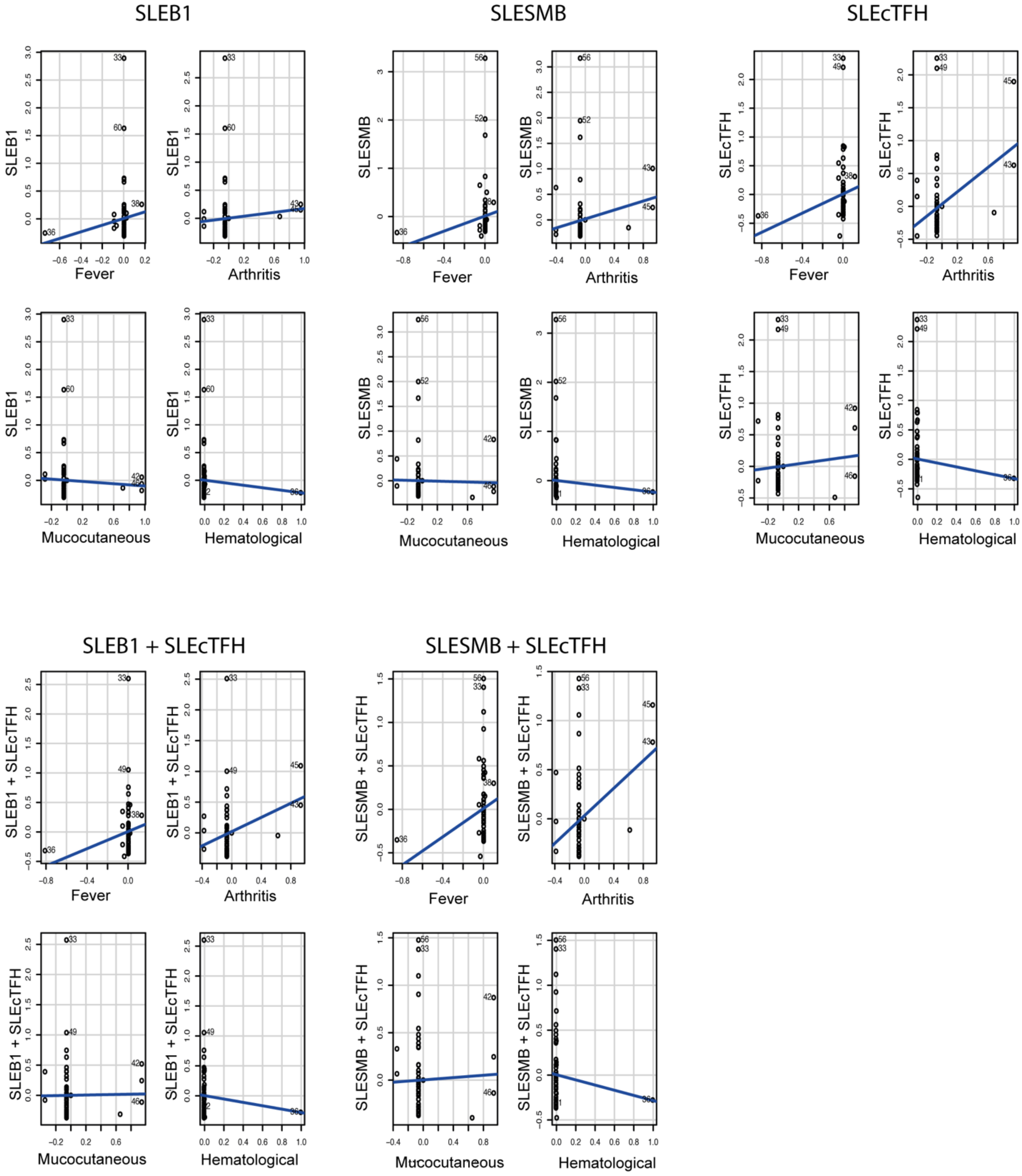

Supplementary Figure 9. Multivariate linear regression of cell subsets of interest and clinical features. Multivariate linear regression analysis between cell subset measurements (top: SLEB1, SLESMB, SLEcTFH) and clinical features (fever n=1, arthritis n=3, mucocutaneous n=5, hematological n=2), SLESMB-arthritis *p=0.0324. Multivariate linear regression analysis between combined measurements (bottom: SLEB1-SLEcTFH and SLESMB-SLEcTFH) and clinical features (fever n=1, arthritis n=3, mucocutaneous n=5, hematological n=2), SLESMB-SLEcTFH-arthritis *p=0.0316.
